# Astrocyte metabolism of the medium-chain fatty acids octanoic acid and decanoic acid promotes GABA synthesis in neurons via elevated glutamine supply

**DOI:** 10.1186/s13041-021-00842-2

**Published:** 2021-09-03

**Authors:** Jens V. Andersen, Emil W. Westi, Emil Jakobsen, Nerea Urruticoechea, Karin Borges, Blanca I. Aldana

**Affiliations:** 1 Department of Drug Design and Pharmacology, Faculty of Health and Medical Sciences, University of Copenhagen, Universitetsparken 2, 2100 Copenhagen E, Denmark; 2 Department of Pharmacology, School of Biomedical Sciences, The University of Queensland, St. Lucia, Australia

**Keywords:** MCT, MCFA, Neurotransmitter recycling, Mitochondria, β-hydroxybutyrate, Caprylic acid (C8), Capric acid (C10)

## Abstract

The medium-chain fatty acids octanoic acid (C8) and decanoic acid (C10) are gaining attention as beneficial brain fuels in several neurological disorders. The protective effects of C8 and C10 have been proposed to be driven by hepatic production of ketone bodies. However, plasma ketone levels correlates poorly with the cerebral effects of C8 and C10, suggesting that additional mechanism are in place. Here we investigated cellular C8 and C10 metabolism in the brain and explored how the protective effects of C8 and C10 may be linked to cellular metabolism. Using dynamic isotope labeling, with [U-^13^C]C8 and [U-^13^C]C10 as metabolic substrates, we show that both C8 and C10 are oxidatively metabolized in mouse brain slices. The ^13^C enrichment from metabolism of [U-^13^C]C8 and [U-^13^C]C10 was particularly prominent in glutamine, suggesting that C8 and C10 metabolism primarily occurs in astrocytes. This finding was corroborated in cultured astrocytes in which C8 increased the respiration linked to ATP production, whereas C10 elevated the mitochondrial proton leak. When C8 and C10 were provided together as metabolic substrates in brain slices, metabolism of C10 was predominant over that of C8. Furthermore, metabolism of both [U-^13^C]C8 and [U-^13^C]C10 was unaffected by etomoxir indicating that it is independent of carnitine palmitoyltransferase I (CPT-1). Finally, we show that inhibition of glutamine synthesis selectively reduced ^13^C accumulation in GABA from [U-^13^C]C8 and [U-^13^C]C10 metabolism in brain slices, demonstrating that the glutamine generated from astrocyte C8 and C10 metabolism is utilized for neuronal GABA synthesis. Collectively, the results show that cerebral C8 and C10 metabolism is linked to the metabolic coupling of neurons and astrocytes, which may serve as a protective metabolic mechanism of C8 and C10 supplementation in neurological disorders.

**Supplementary Information:**

The online version contains supplementary material available at 10.1186/s13041-021-00842-2.

## Introduction

Glucose is the primary fuel of the brain. However, brain cells can utilize several other metabolic substrates including amino acids, ketone bodies and fatty acids [1]. Medium-chain fatty acids (MCFAs) are gaining attention as brain fuels [2–4]. Diets enriched with medium-chain triglycerides (MCTs) of octanoic acid (C8) and decanoic acid (C10) have shown beneficial effects in neurodegenerative diseases [5]. In patients with mild cognitive impairment and Alzheimer’s disease, supplementation of C8 and C10 has been shown to improve both cognition and brain energy metabolism [6–8]. Furthermore, MCT supplementation appears to be efficient in the management of drug-resistant epilepsy [9–11]. Ketone body production driven by hepatic metabolism of C8 and C10 has been suggested to be the main mechanism underlying the anti-epileptic effects of the MCT diet [9]. However, studies have found poor correlation between seizure control and plasma ketone levels [12, 13]. Both C8 and C10 are able to cross the blood brain barrier [14, 15] and can reach cerebral concentrations of 200 µM in mice after enriched feeding [16, 17]. As C8 and C10 are well-known metabolic substrates in the periphery [2], they may serve specific metabolic purposes in the brain as well.

Neurons and astrocytes function in close metabolic collaboration [18]. The energy metabolism of neurons and astrocytes is closely linked to both synthesis and metabolism of glutamate and GABA, being the primary excitatory and inhibitory neurotransmitters of the brain, respectively [19, 20]. Astrocytes take up a large fraction of the released glutamate and GABA from the synapse, and in turn, provide neurons with glutamine, which is an essential substrate for re-synthesis of glutamate and GABA in the neurons [21, 22]. Inadequate astrocyte glutamine synthesis has been reported in multiple diseases, including epilepsy, and experimental inhibition of glutamine synthesis leads to seizures [23–25]. Glutamine is exclusively synthesized in astrocytes from glutamate catalyzed by the enzyme glutamine synthetase (GS) [26]. This process is closely linked to cellular energy metabolism as the tricarboxylic acid (TCA) cycle intermediate α-ketoglutarate serves as the precursor of glutamate synthesis and hereby also glutamine synthesis in astrocytes [27]. In the mitochondria, C8 and C10 are converted by β-oxidation into acetylCoA units, which can enter the TCA cycle and hereby support cellular metabolism. Previous studies have suggested that astrocytes may be the main metabolic compartment for C8 metabolism in the brain [28–30]. However, brain metabolism of C8 and C10 and how this may be linked to neurotransmitter recycling, has not been explored in detail.

The aim of this study was therefore to provide a better understanding of cellular C8 and C10 metabolism in the brain. To achieve this we mapped C8 and C10 metabolism in acutely isolated cerebral cortical slices by using ^13^C isotopically enriched C8 and C10 with subsequent gas chromatography-mass spectrometry (GC–MS) analysis. We also conducted metabolic competition assays between C8 and C10 and investigated the effects of carnitine palmitoyltransferase I (CPT-1) and GS inhibition. Furthermore, we examined the effect of C8 and C10 supplementation on mitochondrial bioenergetics in cultured astrocytes. We provide evidence that both C8 and C10 are oxidatively metabolized in brain slices particularly promoting astrocyte glutamine synthesis. Notably, we demonstrate that the glutamine generated by C8 and C10 metabolism in astrocytes is utilized in neurons for GABA synthesis. Our results provide new insight into C8 and C10 metabolism in the brain and potential metabolic mechanisms of MCFA supplementation in different pathologies.

## Methods

### Materials

The stable ^13^C enriched compounds [U-^13^C]octanoic acid ([U-^13^C]C8, CLM-3981-PK, 98%), [U-^13^C]decanoic acid ([U-^13^C]C10, CLM-9950-PK, 98%) and [U-^13^C]β-hydroxybutyrate ([U-^13^C]βHB, CLM-3853-PK, sodium salt, 97%) were all from Cambridge Isotope Laboratories (Tewksbury, USA). Octanoic acid (^12^C8, C2875), decanoic acid (^12^C10, C1875), (*R*)-β-hydroxybutyrate (^12^C-βHB, 54920) oligomycin A (75351), carbonyl cyanide 4-(trifluoromethoxy)phenylhydrazone (FCCP, C2920), antimycin A (A8674) rotenone (R8875), (+)-etomoxir (E1905, sodium salt) and l-methionine sulfoximine (MSO, M5379) were from Sigma-Aldrich (St. Louis, MO, USA). All other chemicals used were of the purest grade available from regular commercial sources.

### Animals

Male NMRI mice (Envigo, Cambridgeshire, United Kingdom) of 12 weeks of age (weight: 40.5 ± 0.5 g) were housed in a pathogen-free, temperature and humidity controlled environment at the Department of Drug Design and Pharmacology, University of Copenhagen. The mice were acclimatized for two weeks before experiments and were single-housed in individually ventilated cages with free access to chow and water.

### Brain slice incubations

Incubation of acutely isolated cerebral cortical mouse brain slices were performed as previously described [31], with slight modifications. Briefly, a mouse was euthanized by cervical dislocation and decapitated. The brain was transferred to ice-cold artificial cerebrospinal fluid (ACSF) containing in mM: NaCl 128, NaHCO_3_ 25, d-glucose 10, KCl 3, CaCl_2_ 2, MgSO_4_ 1.2, KH_2_PO_4_ 0.4, pH = 7.4. The cerebral cortex was dissected and sliced (350 µm) using a McIlwain tissue chopper (The Vibratome Company, O’Fallon, MO, USA). The cerebral cortical slices were kept just below the surface of 10 mL 37 °C oxygenated (5% CO_2_/95% O_2_) ACSF and pre-incubated for 60 min. Subsequently, the media were exchanged for ACSF containing the stable ^13^C enriched compounds (12 mL in glass tubes to avoid fatty acid adherence to plastic) supplemented with 5 mM unlabeled d-glucose and incubated for additional 60 min. Two round of slices incubation experiments were performed. In the first round, a competition assay was performed in which the slices were incubated in the presence of either [U-^13^C]C8, [U-^13^C]C10 or [U-^13^C]βHB (all 200 µM) ± competing unlabeled ^12^C substrates: ^12^C8, ^12^C10 or ^12^C-βHB (also 200 µM). Media containing C10 (^12^C/^13^C) was prepared directly in ACSF at a final concentration of 200 µM, whereas C8 (^12^C/^13^C) and βHB (^12^C/^13^C) were prepared as 20 mM stocks. C8/C10 at concentrations of 200 µM have previously been found sufficient to support cellular metabolism [32], and further allows dissolution without the addition of aprotic solvents e.g. dimethylsulfoximide (DMSO) which impairs cellular metabolism [33]. In the second round, the effects of the two metabolic inhibitors etomoxir (stock: 5 mM, final concentration: 100 µM) and MSO (stock: 200 mM, final concentration: 5 mM) were investigated. To ensure efficient inhibition, prior to the addition of the ^13^C enriched substrates, the inhibitors were applied after 30 min of pre-incubation and also throughout the entire incubation period. All incubations were terminated by transferring slices into ice-cold 70% ethanol. The slices were sonicated and centrifuged (4000*g* × 20 min) and the supernatant was removed and lyophilized before GC–MS or HPLC analysis. Pellets were saved for protein determination by Pierce protein assay.

### Metabolic mapping using gas chromatography–mass spectrometry (GC–MS) analysis

[U-^13^C]C8, [U-^13^C]C10 and [U-^13^C]βHB will all enter cellular metabolism as ^13^C enriched acetylCoA, which will lead to ^13^C enrichment of TCA cycle intermediates and connected amino acids. The metabolite ^13^C enrichment from metabolism of [U-^13^C]C8, [U-^13^C]C10 and [U-^13^C]βHB was determined by GC–MS analyses as previously described [34]. Briefly, slice extracts were reconstituted in water, acidified, extracted twice with ethanol and the metabolites were derivatized using *N*-tert-butyldimethylsilyl-*N*-methyltrifluoroacetamide. Samples were analyzed by GC (Agilent Technologies, 7820A, J&W GC column HP-5 MS) coupled to MS (Agilent Technologies, 5977E). The isotopic enrichment was corrected for the natural abundance of ^13^C by analyzing standards of the unlabeled metabolites of interest. Metabolism of ^13^C enriched substrates can give rise to complex labeling patterns with multiple ^13^C isotopologues which are metabolites differing in their ^13^C composition (denoted as M + X, where M is the molecular ion of the metabolite and X is the number is ^13^C atoms) as described in [34]. Here data is presented as the molecular carbon labeling (MCL), which is the weighted average of all the different isotopologues of a metabolite [35]. The MCL thus provides a measurement of the overall ^13^C accumulation in a given metabolite and is calculated as:

$$Molecular \, carbon \, labeling \left( {MCL} \right) = \frac{{\left( {M + 1*1} \right) + \left( {M + 2*2} \right) + \left( {M + 3*3} \right)...\left( {M + X*X} \right)}}{Total \, number \, of \, carbon \, atoms \, in \, molecule}$$

### Determination of amino acid amounts by high-performance liquid chromatography (HPLC) analysis

Aqueous extracts of the brain slices were analyzed by reverse-phase HPLC (Agilent Technologies, 1260 Inifinity, Agilent ZORBAX Eclipse Plus C18 column) to quantitatively determine the amounts of the following amino acids: aspartate, glutamate, glutamine, taurine & GABA [36]. A pre-column derivatization with o-phthalaldehyde and fluorescent detection, λ_ex_ = 338 nm, λ_em_ = 390 nm, was performed. Gradient elution with mobile phase A (10 mM NaH_2_PO_4_, 10 mM Na_2_B_4_O_7_, 0.5 mM NaN_3_, pH 8.2) and mobile phase B (acetonitrile 45%: methanol 45%: H_2_O 10%, V:V:V) was performed. The amounts of amino acids were determined from analysis of standards of the amino acids of interest. The amino acid amounts of the slice extracts were adjusted to the protein amounts of the slices and are presented as nmol/mg protein.

### Primary cultures of cortical astrocytes and determination of oxygen consumption rate (OCR)

Primary cultures of cortical astrocytes were prepared from 7-day-old NMRI mouse pups as previously described [34]. Each plate of cortical astrocytes were prepared from individual batches of pups. Briefly, pups were decapitated and the cerebral cortex excised. The tissue was processed by passage through a 80 μm pore size nylon mesh and submersed in a modified Dulbecco’s Modified Eagle’s Medium (DMEM) supplemented with 2.5 mM l-glutamine, 6 mM glucose, 100,000 i.u. penicillin, 0.04 mM phenol red, 26.2 mM NaHCO_3_ and 20% (v/v) fetal bovine serum. A steel cannula fitted to a syringe was used to dissociate the cells by trituration. The cell suspension was plated in Seahorse XFe96-well culture plates and cultured for 21 days at 37 °C in a humidified atmosphere of 5% CO_2_/95% air. Medium was exchanged twice a week and serum concentration reduced to 15% in the second week and 10% in the third week and 0.25 mM dibutyryl-cAMP (final concentration) was added to the medium for the last week of culture. The oxygen consumption rate (OCR) of the cultured astrocytes were determined using a Seahorse XFe96 analyzer as previously described [37]. Briefly, the astrocytes were washed twice and the media changed to Seahorse assay medium (DMEM with 2.5 mM glucose and 3 mg/L phenol red, pH 7.4) and kept at 37 °C (non-CO_2_ incubator) for 1 h. When indicated the assay medium was further supplemented with C8, C10 or the combination (all 200 µM). As for the brain slice incubations, media containing C10 was prepared directly in Seahorse assay medium at a final concentration of 200 µM, whereas C8 was prepared as a 20 mM stock. In total, 12 measurement cycles (2 min mix, 1 min wait, 3 min measure) were performed (as outlined in Fig. 2B). After 3 initial baseline measurements, 3 injections were performed (all final concentrations): (1) 1.0 µM oligomycin A, (2) 0.5 µM FCCP, (3) 0.5 µM antimycin A and 0.5 µM rotenone. After each injection 3 measurement cycles were performed. The non-mitochondrial OCR was subtracted from all data points (Fig. 2B). To account for potential inter-well variability during the culturing period, data is presented as OCR as % of control (glucose condition).

### Experimental design and statistical analyses

Data is presented as means ± standard error of the mean (SEM), with individual data points presented. Each data point (represented by circles in the graphs) represents biological replicates (i.e. from individual animals), which is denoted by ‘n’ in the figure legends. Statistical analyses were performed by repeated measurements 1-way ANOVA with Bonferroni correction for multiple comparisons. The significance level was set at p < 0.05 and is indicated with a single asterisk.

## Results

### Oxidative C8 and C10 metabolism promotes astrocyte glutamine synthesis in brain slices

**Fig. 1:**
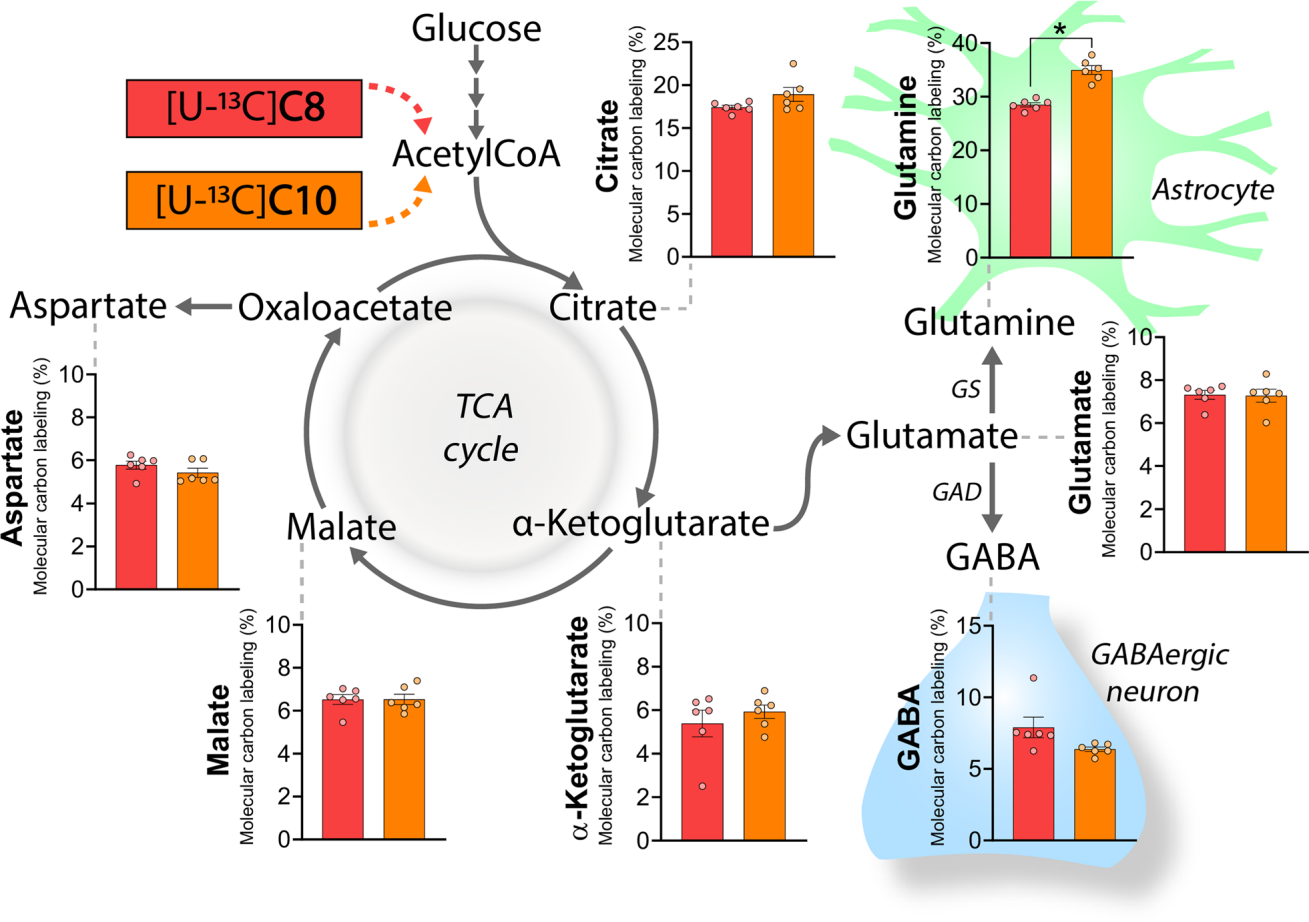
The extent of C8 and C10 metabolism is comparable and promotes astrocyte glutamine synthesis in brain slices. Metabolism of [U-^13^C]C8 (red bars) and [U-^13^C]C10 (orange bars) in mouse cerebral cortical slices. [U-^13^C]C8 and [U-^13^C]C10 can undergo β-oxidation and hereby enter the TCA cycle as ^13^C acetylCoA. The entry of ^13^C acetylCoA will give rise to ^13^C accumulation in TCA cycle intermediates and amino acids (presented as the weighted average of ^13^C accumulation, molecular carbon labeling, MCL). [U-^13^C]C8 and [U-^13^C]C10 were provided at a concentration of 200 µM in addition to 5 mM d-glucose. Glutamate can be converted to glutamine by glutamine synthetase (GS) selectively expressed in astrocytes. In GABAergic neurons, glutamate can be converted to GABA by glutamate decarboxylase (GAD) activity. C8: octanoic acid. C10: decanoic acid. Mean ± SEM, n = 6 from individual animals, repeated measures 1-way ANOVA with Bonferroni post hoc test, *p < 0.05

To get an overview of the extent and possible compartmentation of C8 and C10 brain metabolism, we first incubated acutely isolated cerebral cortical slices in the presence of either [U-^13^C]C8 or [U-^13^C]C10 (Fig. 1). [U-^13^C]C8 and [U-^13^C]C10 will enter cellular metabolism as ^13^C enriched acetylCoA units and hereby give rise to ^13^C enrichment in TCA cycle intermediates and derived amino acids (presented as the average ^13^C accumulation, molecular carbon labeling, MCL [35]). In the TCA cycle, the highest ^13^C accumulation from both [U-^13^C]C8 and [U-^13^C]C10 metabolism was found in citrate (C8: 17.4 ± 0.2%, C10: 18.9 ± 0.8%), whereas it was much lower for α-ketoglutarate (C8: 5.4 ± 0.6%, C10: 5.9 ± 0.3%) and malate (C8: 6.5 ± 0.2%, C10: 6.5 ± 0.2%). Of the derived amino acids, the highest ^13^C accumulation was found in glutamine (C8: 28.5 ± 0.4%, C10: 35.0 ± 0.8%), whereas it was around four times lower for aspartate (C8: 5.8 ± 0.2%, C10: 5.4 ± 0.2%), glutamate (C8: 7.3 ± 0.2%, C10: 7.3 ± 0.3%) and GABA (C8: 7.9 ± 0.7%, C10: 6.4 ± 0.2%). The only difference between the two fatty acids was observed in glutamine, where metabolism of [U-^13^C]C10 led to larger ^13^C accumulation than [U-^13^C]C8. We found no differences in the absolute pool sizes of amino acids between the [U-^13^C]C8 and [U-^13^C]C10 incubations (Additional file 1: Table S1). These results show that both C8 and C10 are actively metabolized in brain slices and that the extent of metabolism between the two fatty acids is generally comparable. Furthermore, since glutamine is selectively synthesized in astrocytes [26], the preferential ^13^C accumulation in this amino acid suggesting that astrocytes are the primary cellular compartment of C8 and C10 metabolism in brain slices.

### C8 and C10 stimulate mitochondrial respiration in cultured astrocytes

**Fig. 2:**
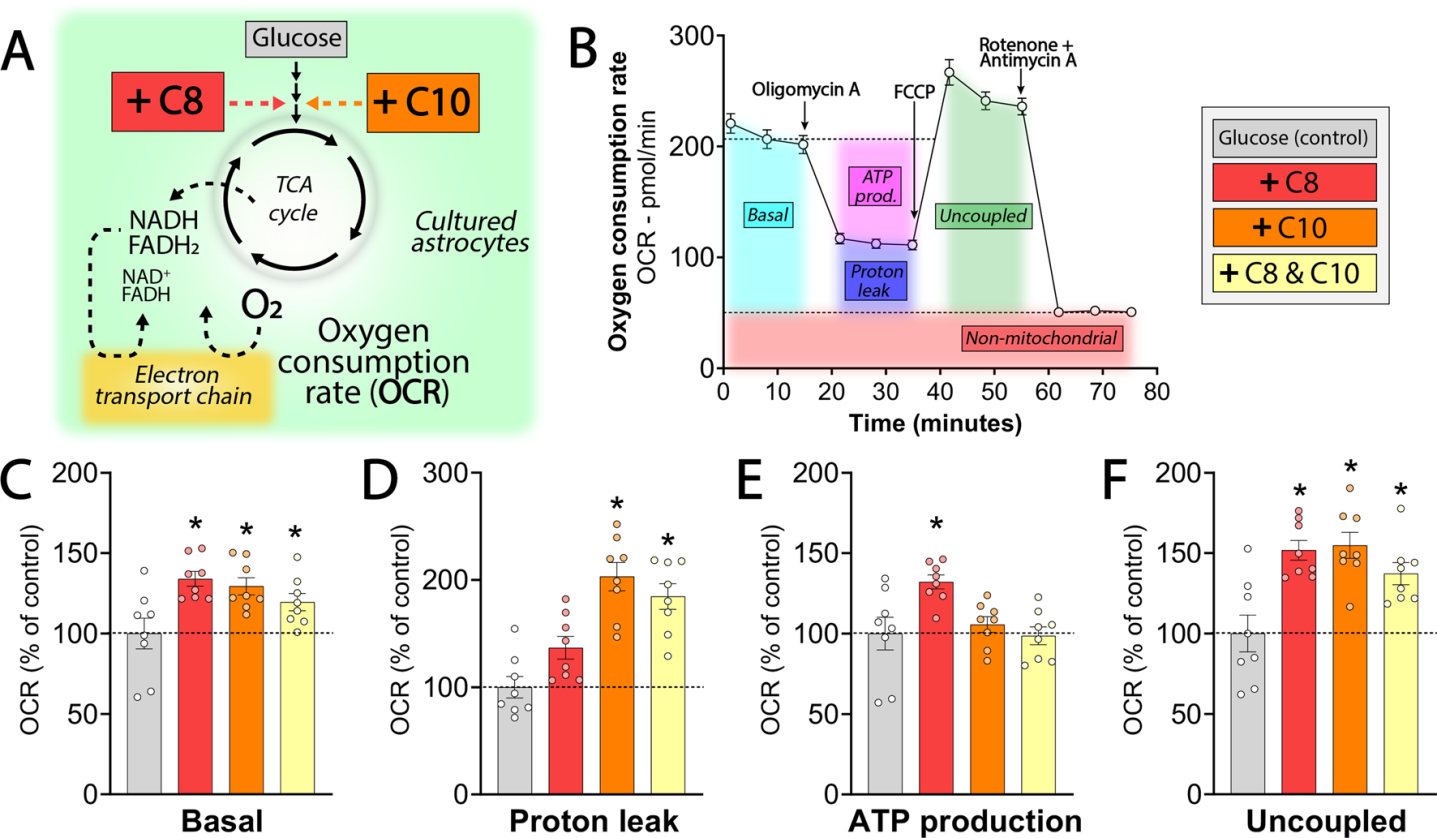
C8 and C10 stimulate mitochondrial respiration in cultured astrocytes via distinct mechanisms. **A** Cultured astrocytes were provided C8, C10 or the combination and the mitochondrial oxygen consumption rate (OCR) was assessed. The astrocytes were pre-incubated with the MCFAs (200 µM) for 1 h in addition to 2.5 mM d-glucose. **B** Seahorse XFe96 assay overview. During the assay three compounds were injected: (1) the ATP synthase inhibitor oligomycin A, (2) the mitochondrial uncoupler FCCP, (3) the electron transport chain inhibitors rotenone and antimycin A. From the Seahorse assay multiple respiratory parameters can be assessed: non-stimulated OCR (Basal), mitochondrial proton leak, OCR related to ATP production (ATP prod.), maximal uncoupled OCR (uncoupled) and the OCR unrelated to mitochondrial processes (non-mitochondrial). The non-mitochodrial OCR was subtracted from all data points prior to analysis. **C**–**F** Respiratory parameters of cultured astrocytes provided C8 (red bars), C10 (orange bars) or the combination (yellow bars). Data is presented as OCR, relative to the OCR of glucose alone (grey bars, control)). C8: octanoic acid. C10: decanoic acid. Mean ± SEM, n = 8 from individual batches of cultures, repeated measures 1-way ANOVA with Bonferroni post hoc test, *p < 0.05

MCFAs can serve as metabolic substrates and support mitochondrial respiration in multiple tissues [2, 4]. To assess whether this was the case in astrocytes, we pre-incubated cultured astrocytes in media supplemented with C8, C10 or the combination in addition to glucose (Fig. 2A) and determined the oxygen consumption rate (OCR) using the Seahorse XFe96 flux analyzer (Fig. 2B). We found that C8, C10 and the combination increased the basal, i.e. non-stimulated, OCR of the cultured astrocytes (Fig. 2C). The basal OCR is comprised of the OCR related to ATP production and the proton leak (Fig. 2B), the latter reflecting the flow of protons across the mitochondrial membrane not linked to ATP production [38]. We found that the presence of C10 selectively elevated the proton leak in the cultured astrocytes (Fig. 2D), whereas C8 alone increased the OCR related to ATP production (Fig. 2E). Finally, when the oxidative capacity was unleashed by the uncoupling agent FCCP, we observed that C8, C10 as well as the combination, elevated the OCR of the cultured astrocytes (Fig. 2F). These results demonstrate that both C8 and C10 are able to stimulate respiration in cultured astrocytes, albeit via distinct mitochondrial mechanisms.

### C10 is preferred over C8 as a metabolic substrate in brain slices

**Fig. 3:**
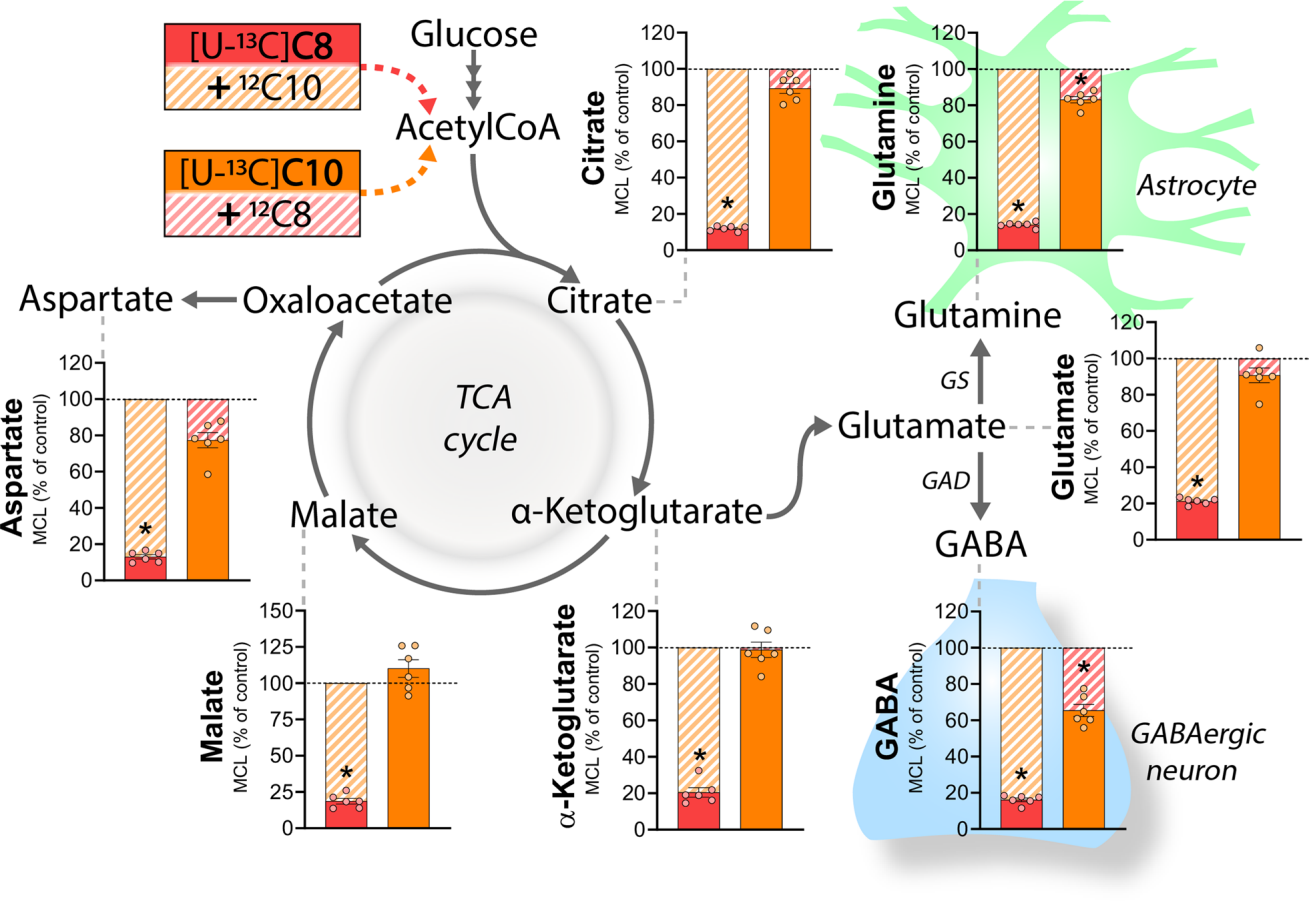
C10 is preferred over C8 as a metabolic substrate in brain slices. Competition assay between [U-^13^C]C8 + unlabeled ^12^C10 and [U-^13^C]C10 + unlabeled ^12^C8 in mouse cerebral cortical slices (molecular carbon labeling, MCL, relative to the MCL without competing ^12^C fatty acids (control)). All MCFAs were provided at concentrations of 200 µM in addition to 5 mM d-glucose. Metabolism of an unlabeled substrate (^12^C) will dilute the ^13^C accumulation and hereby lead to a reduction in the MCL. C8: octanoic acid. C10: decanoic acid, GAD: glutamate decarboxylase, GS: glutamine synthetase. Mean ± SEM, n = 6 from individual animals, repeated measures 1-way ANOVA with Bonferroni post hoc test, *p < 0.05

So far, we have shown that C8 and C10 can serve as metabolic substrates in astrocytes promoting glutamine synthesis and stimulating mitochondrial respiration. Next, we wanted to investigate if there was any metabolic preference between the two fatty acids. To this end, we performed a competition assay, in which brain slices were incubated in the presence of either [U-^13^C]C8 or [U-^13^C]C10, but now also with a competing unlabeled (i.e. ^12^C) fatty acid (Fig. 3). Metabolism of an unlabeled ^12^C fatty acid will dilute the ^13^C labeling, and so the metabolic preference of each fatty acid can be determined. When the slices were exposed to [U-^13^C]C8 in the presence of unlabeled ^12^C10, we found large reductions in the ^13^C accumulation of all measured metabolites (in the range of 12–21% of control). In contrast, the metabolism of [U-^13^C]C10 was only found to be reduced in GABA (65.4 ± 3.4% of control) and glutamine (83.1 ± 1.7% of control) when unlabeled ^12^C8 was present. These results demonstrate that metabolism of C10 is predominant over that of C8 in the brain slices when the two fatty acids are provided together. Furthermore, we also included the ketone body β-hydroxybutyrate (βHB) in the competition experiments. βHB also enters metabolism as acetylCoA units, but is oxidized to a larger extent in neurons than in astrocytes [29, 39]. We found that unlabeled ^12^C-βHB had little influence on both [U-^13^C]C8 and [U-^13^C]C10 metabolism in the brain slices (Additional file 1: Fig. S1). Furthermore, metabolism of unlabeled ^12^C8 and ^12^C10 was only able to reduce the ^13^C accumulation from [U-^13^C]βHB metabolism in citrate and glutamine (Additional file 1: Fig. S2), confirming that the substrates are oxidized in separate cellular compartments. Again, no changes in the absolute pool sizes of amino acids were observed (Additional file 1: Table S1).

### Metabolism of C8 and C10 is independent of carnitine palmitoyltransferase I in brain slices

**Fig. 4:**
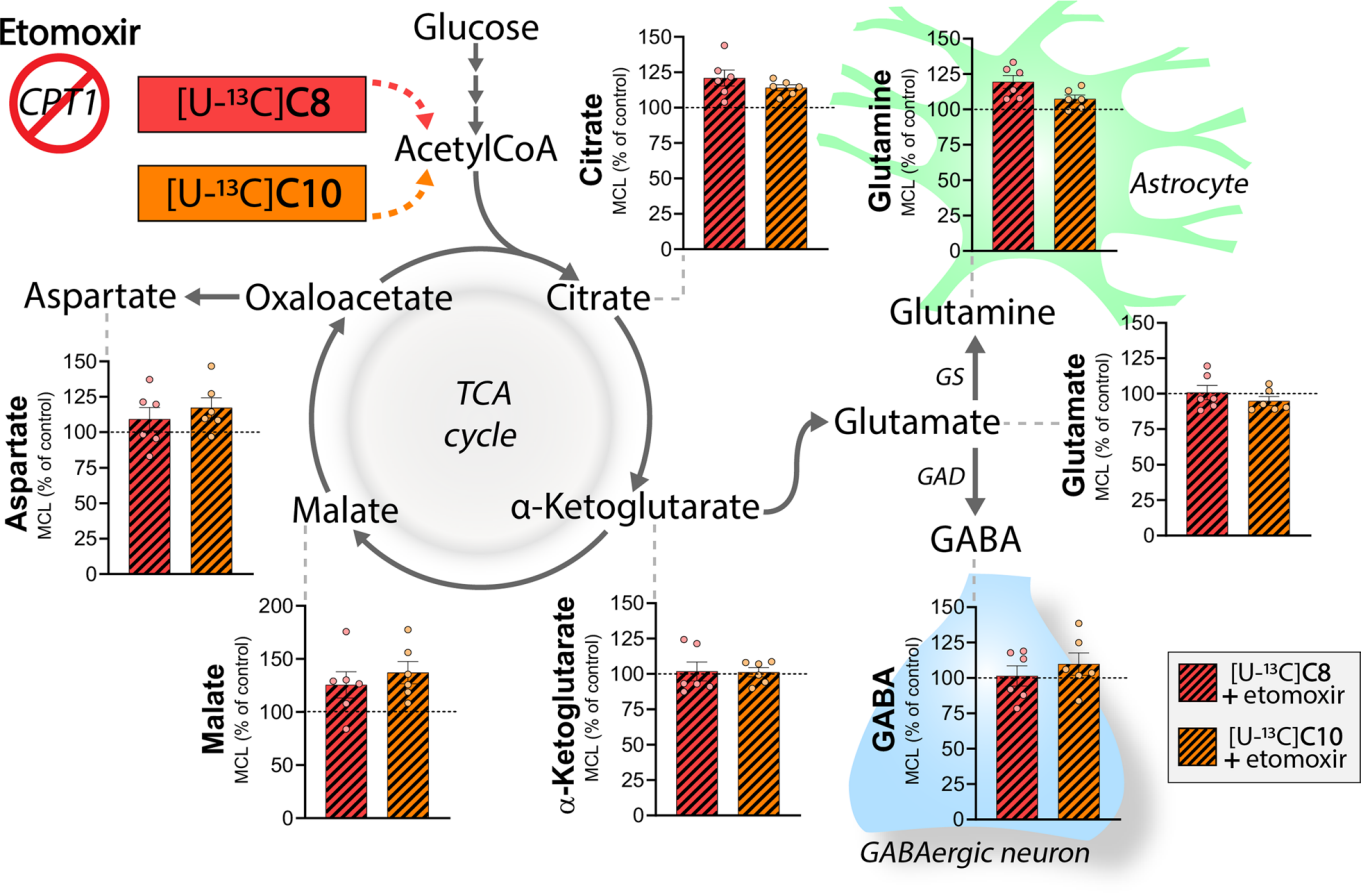
Metabolism of C8 and C10 is independent of carnitine palmitoyltransferase I (CPT1) in brain slices. Metabolism of [U-^13^C]C8 (red/black striped bars) and [U-^13^C]C10 (orange/black striped bars) in the presence of the CPT1 inhibitor etomoxir in mouse cerebral cortical slices (molecular carbon labeling, MCL, relative to the MCL without etomoxir (control)). [U-^13^C]C8 and [U-^13^C]C10 were provided at a concentration of 200 µM in addition to 5 mM d-glucose. Etomoxir was applied at 100 µM. C8: octanoic acid. C10: decanoic acid, GAD: glutamate decarboxylase, GS: glutamine synthetase. Mean ± SEM, n = 6 from individual animals, repeated measures 1-way ANOVA with Bonferroni post hoc test, *p < 0.05

Given that the extent of ^13^C accumulation between C8 and C10 was comparable when metabolized individually (Fig. 1), it was surprising that C10 was preferred over C8 so extensively when applied together (Fig. 3). This could be explained by C8 and C10 competing for the same transport mechanism. Cellular and mitochondrial uptake of MCFAs is driven by passive diffusion [2, 40]. However, in a neuronal-like cell line mitochondrial uptake of C8 and C10 was recently suggested to be partially mediated by carnitine palmitoyltransferase I (CPT-1), an enzyme important for long-chain fatty acid transport [41]. To investigate if this was the case in brain slices, we next examined the effect of the irreversible CPT-1 inhibitor etomoxir on metabolism of [U-^13^C]C8 and [U-^13^C]C10 (Fig. 4). Etomoxir was applied at 100 µM, which effectively inhibit long-chain fatty acid metabolism in both dissected brain tissue and cultured astrocytes [42]. We did not observe any significant alterations in ^13^C accumulation of the measured metabolites when etomoxir was present during the incubation. These results indicate that metabolism of both [U-^13^C]C8 and [U-^13^C]C10 is independent of CPT-1 in brain slices, and C8 and C10 must therefore enter the mitochondria via other mechanisms. As expected, metabolism of [U-^13^C]βHB was likewise unaffected by etomoxir (Additional file 1: Fig. S3).

### Astrocyte glutamine from C8 and C10 metabolism is utilized for neuronal GABA synthesis

**Fig. 5:**
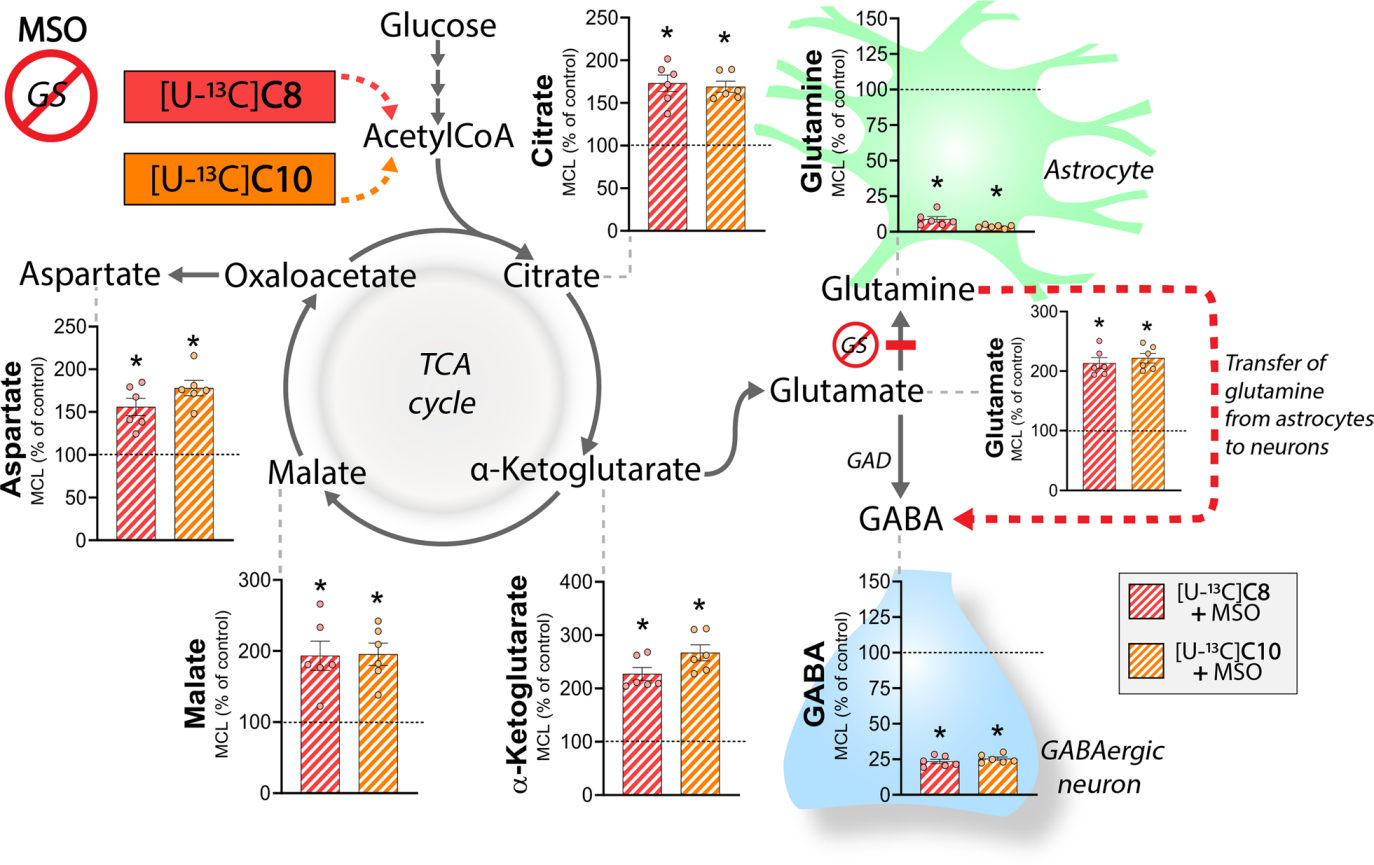
Astrocyte glutamine derived from metabolism of C8 and C10 is utilized for GABA synthesis in neurons. Metabolism of [U-^13^C]C8 (red/white striped bars) and [U-^13^C]C10 (orange/white striped bars) in the presence of the glutamine synthetase (GS) inhibitor MSO in mouse cerebral cortical slices (molecular carbon labeling, MCL, relative to the MCL without MSO (control)). [U-^13^C]C8 and [U-^13^C]C10 were provided at a concentration of 200 µM in addition to 5 mM d-glucose. MSO was applied at 5 mM. C8: octanoic acid. C10: decanoic acid, GAD: glutamate decarboxylase. Mean ± SEM, n = 6 from individual animals, repeated measures 1-way ANOVA with Bonferroni post hoc test, *p < 0.05

To further explore the significance of the astrocyte glutamine synthesis from metabolism of C8 and C10, we finally investigated the effect of glutamine synthetase (GS) inhibition on metabolism of [U-^13^C]C8 and [U-^13^C]C10 in the cerebral cortical slices (Fig. 5). The GS inhibitor methionine sulfoximine (MSO) was applied at 5 mM, which we have previously shown is sufficient to effectively inhibit GS activity in brain slices [43]. As expected, GS inhibition abolished the ^13^C accumulation in glutamine from metabolism of [U-^13^C]C8 and [U-^13^C]C10 (C8: 8.7 ± 1.9% of control, C10: 3.7 ± 0.5% of control). Interestingly, all TCA cycle intermediates and the derived amino acids aspartate and glutamate exhibited large increases in ^13^C accumulation from metabolism of [U-^13^C]C8 and [U-^13^C]C10 when glutamine synthesis was inhibited by MSO (in the range of 156–267% of control). In contrast, GS inhibition selectively led to large reductions in the ^13^C accumulation in GABA (C8: 23.4 ± 1.5% of control, C10: 25.4 ± 1.2% of control) from both [U-^13^C]C8 and [U-^13^C]C10 metabolism. Since astrocyte-derived glutamine is a crucial substrate for neuronal GABA synthesis [44], these results demonstrate that the glutamine synthesized by metabolism of C8 and C10 in astrocytes is utilized in neurons to maintain the GABA pool. MSO only decreased the ^13^C accumulation in glutamine, but not in GABA, from [U-^13^C]βHB metabolism which is expected as βHB is primarily metabolized in neurons (Additional file 1: Fig. S4).

## Discussion

**Fig. 6:**
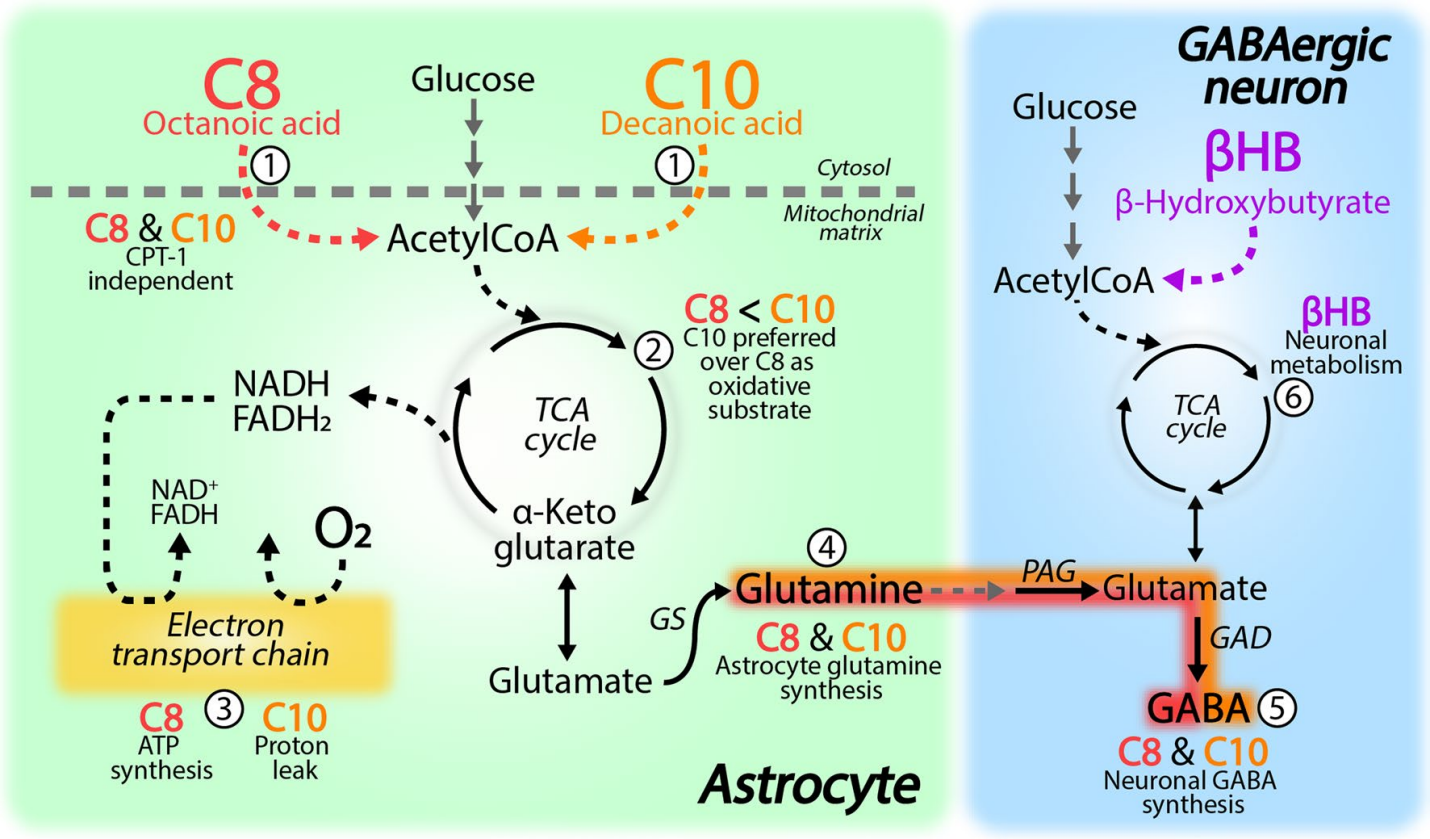
Overview of C8 and C10 metabolism in brain slices. (**1**) Both C8 and C10 metabolism was unaffected by the carnitine palmitoyltransferase I (CPT-1) inhibitor etomoxir. (**2**) When provided alone, the extent of C8 and C10 metabolism was comparable, but when provided together C10 was preferred over C8 as a metabolic substrate. (**3**) In cultured astrocytes, C8 selectively increased the respiration linked to ATP production, whereas the presence of C10 elevated the mitochondrial proton leak. (**4**) Both C8 and C10 is utilized for glutamine synthesis. Since glutamine is selectively synthesized in astrocytes, this signifies that astrocytes are the primary cellular compartment for C8 and C10 metabolism. (**5**) When glutamine synthesis from C8 and C10 metabolism was inhibited, a selective reduction was observed in neuronal GABA synthesis. This demonstrates that the glutamine generated by astrocyte metabolism of C8 and C10 is used for neuronal GABA synthesis. (**6**) The ketone body β-hydroxybutyrate (βHB) did not compete, to any significant extent, with C8 and C10 metabolism supporting the notion that ketone bodies are primarily utilized by neurons

Here we demonstrate that the MCFAs C8 and C10 are oxidatively metabolized in brain slices and promotes astrocyte glutamine synthesis. We found that the overall extent of metabolism was comparable between C8 and C10, but C10 was preferred over C8 as a metabolic substrate when both fatty acids were present. Both C8 and C10 stimulated mitochondrial respiration in cultured astrocytes, where C8 elevated respiration linked to ATP synthesis and C10 increased the mitochondrial proton leak. Finally, we found that metabolism of both C8 and C10 was CPT-1 independent and that the elevated astrocyte glutamine synthesis from C8 and C10 metabolism is utilized for neuronal GABA synthesis in the brain slices (findings summarized in Fig. 6).

### Astrocyte metabolism of C8 and C10

MCFAs have long been known to be substrates, albeit minor ones, for cerebral oxidative metabolism [1, 4, 45]. It has previously been reported that in-vivo metabolism of radiolabeled C8 leads to a larger enrichment in glutamine than in glutamate, suggesting a preferential astrocyte metabolism of C8 [28, 30]. Here we extend this finding to acute brain slices, where we show that metabolism not only of C8, but also of C10, is promoting glutamine synthesis in astrocytes. The preferential astrocyte metabolism of MCFAs has further been confirmed in cell cultures, where only astrocytes were able to metabolize [1-^14^C]C8, whereas neurons and oligodendrocytes were not [29]. Here we also report that the extent of C8 and C10 metabolism in brain slices, based on ^13^C accumulation in TCA cycle intermediates and amino acids, was generally comparable. A recent study assessed CO_2_ generation from metabolism of [1-^14^C]C8/C10 in the neuronal-like SH-SY5Y cell line. Based on the CO_2_ generation, the authors reported that the oxidation of C8 was around 6 times higher than that of C10 [41], which is in contrast to our findings in the acute brain slices where the extent of C8 and C10 metabolism was comparable (Fig. 1). This discrepancy is likely explained by the different preparations, as the SH-SY5Y is a neuroblastoma cell line with neuronal-like properties [46], whereas brain slices are a complex preparation containing all types of cerebral cells, including the primary cells of MCFA metabolism in the brain, the astrocytes. When the brain slices were provided both C8 and C10, we found that metabolism of C10 was predominant over that of C8 (Fig. 3), which may indicate competition in mitochondrial transport or metabolism of C8 and C10. It has also recently been reported that CPT-1 inhibition reduced the oxidative metabolism of both C8 and C10 in the SH-SY5Y cells [41]. This is surprising as MCFAs metabolism should be carnitine independent [2, 47], which we also found to be the case in brain slices (Fig. 4). Our observation that C10 was preferred over C8 as a metabolic substrate in the brain slices may instead be explained by differential substrate affinities for C8 and C10 of the enzymes catalyzing MCFA metabolism. Indeed, the enzyme initiating mitochondrial MCFA β-oxidation, the medium-chain acyl-CoA dehydrogenase, has been shown to have a higher substrate affinity towards C10 than C8 [48]. Furthermore, C10 also has a larger membrane permeability than C8, due to the longer carbon chain, which increases diffusion across the cell and mitochondrial membranes [40], which may also contribute to the observed preferential metabolism of C10 over C8. Although astrocytes are the main cellular compartment of MCFA metabolism, it was recently demonstrated that a subset of neurons in the hypothalamus have a large capacity for C8 oxidation [49]. In these hypothalamic neurons, C8 metabolism regulate neuronal excitability and food intake, which underlines the complexity and importance of regional brain energy metabolism. Further studies are needed to explore other potential functions of MCFA metabolism in the brain, both on a regional and cell-specific level.

### Mitochondrial effects of C8 and C10 in astrocytes

Both C8 and C10 can be utilized as mitochondrial fuels in several different tissues including the brain [2, 4]. Here, we found that the addition of C8, C10 and the combination was able to stimulate both basal and uncoupled mitochondrial respiration in cultured astrocytes (Fig. 2). Our findings are in contrast to a report by Thevenet et al. showing that acute exposure to C8 and C10 was unable to stimulate the OCR of cultured induced pluripotent stem cell-derived human neurons and astrocytes [50]. Although not significant, the study reported that C8 had a tendency to increase the overall OCR, whereas C10 had a tendency towards reduced ATP-dependent OCR, in the cultured astrocytes [50]. These tendencies are in line with our findings that C8 increased the respiration linked to ATP production, whereas C10 increased the proton leak in the cultured astrocytes. Our observations further confirm a recent study in cultured astrocytes, showing that both C8 and C10 were able to increase basal respiration, whereas C10 selectively increased the proton leak relative to pyruvate oxidation [32]. The proton leak is defined as the flow of protons across the inner mitochondrial membrane unrelated to ATP synthesis and is intimately coupled to the generation of harmful reactive oxygen species (ROS) [38]. Intriguingly, a mild increase in the proton leak is protective against ROS generation [38, 51] and the elevated proton leak induced by C10 may therefore have antioxidant properties. Furthermore, C10 has been shown to exert multiple other antioxidant effects, including regulation of antioxidant gene expression [32], catalase activity [52] and glutathione content [53]. Treatment with C10, but not C8, in SH-SY5Y cells has also been shown to increase mitochondrial biogenesis [52], potentially via PPAR signaling [54, 55]. Collectively these findings show that C10 is not just a mitochondrial fuel, but can exert several beneficial effects on mitochondrial bioenergetics via multiple distinct mechanisms.

### Effects of C8 and C10 metabolism on neuron-astrocyte metabolic coupling and neurotransmitter recycling

Energy and neurotransmitter metabolism in neurons and astrocytes is tightly coupled [18]. Fatty acid transfer between neurons and astrocytes is gaining attention [56, 57] and MCFAs have also been suggested to take part in the transcellular metabolic coupling [3]. Several studies have shown that C10 is able to induce astrocyte lactate production, whereas C8 stimulates astrocyte ketone body production [50, 53, 58, 59], both of which may be transferred to neurons to support oxidative metabolism. Another crucial metabolite transferred from astrocytes to neurons is glutamine, which serves an essential role as the primary precursor of neuronal glutamate and GABA synthesis [20–22, 44]. When we inhibited glutamine synthesis from C8 and C10 metabolism in brain slices using MSO, the ^13^C accumulation in glutamine and GABA was decreased, while it was elevated for the rest of the measured metabolites (Fig. 5). We have previously described similar effects of MSO on metabolism of the well-known astrocytic substrate [1,2-^13^C]acetate [43], further supporting that astrocytes are the main cellular compartment of C8 and C10 metabolism. Furthermore, we have previously demonstrated that transfer of astrocyte-derived glutamine to neurons is maintained in our slice incubation set-up [43, 60], implying that the decreased neuronal GABA synthesis in the presence of MSO is indeed caused by the absent astrocyte glutamine supply. Insufficient astrocyte glutamine synthesis can lead to seizures [24] and reduced expression of GS has been found in the epileptic hippocampus [23]. Elevated neuronal GABA synthesis, linked to increased glutamine supply from astrocyte metabolism of C8 and C10, could therefore aid to maintain the inhibitory tone of the brain and hereby serve as an anti-convulsant mechanism of C8 and C10 supplementation. In addition, Lee et al. recently demonstrated that long-term C10 exposure leads to GABA synthesis and accumulation in cultured astrocytes [59], which may further aid to sustain tonic inhibition [61, 62]. Treatment with MCTs, containing triglycerides of C8 and C10, leads to increased hepatic production of ketone bodies, which has been hypothesized to be the main mechanism behind the beneficial effects in epilepsy [9]. When we provided the ketone body βHB as a competing metabolic substrate to C8 and C10 in brain slices, we observed scarce effects (Additional file 1: Fig. S1 and S2). These observations are in line with the notion that C8 and C10 are primarily metabolized in astrocytes, whereas βHB is metabolized to larger extent in neurons [29, 39]. This clear metabolic compartmentation is highly interesting in relation to dietary MCT treatment, as it will provide specific cellular metabolic substrates; ketone bodies for the neurons to support oxidative metabolism, and C8 and C10 for the astrocytes, which leads to elevated glutamine synthesis (Fig. 6). These notions are in line with a study showing that βHB, but not C8, could support neuronal signaling in hippocampal brain slices during low-glucose conditions [63]. However, the same study reported that C8 increased the neuronal recovery rate [63], which in the light of the present study, could be mediated via increased astrocyte glutamine supply. In addition to direct metabolic effects, C10 may serve several signaling purposes in the brain [59, 64], and is able to directly inhibit AMPA receptors [65], suggesting multiple mechanisms underlying the anti-epileptic effects of C10. Finally, astrocyte dysfunction is gaining attention in several neurodegenerative diseases [66] and we have demonstrated inadequate astrocyte glutamine supply in mouse models of both Alzheimer’s and Huntington’s disease [25, 60]. Interestingly, C8 and C10 supplementation has been shown to improve cognitive function in patients with mild cognitive impairment and Alzheimer’s disease [6–8] and are gaining attention in several other diseases [5]. C8 and C10 could exert these beneficial effects, in part, by enhancing astrocyte glutamine synthesis and hereby improve neuronal neurotransmitter homeostasis. These notions encourage further studies into the molecular and cellular mechanisms of MCFA supplementation in neurodegeneration and other diseases.

## Supplementary Information

**Additional file 1: Figure S1**. Unlabeled ^12^C-βHB competes poorly with [U-^13^C]C8 and [U-^13^C]C10 in brain slices. **Figure S2.** Unlabeled ^12^C8 and ^12^C10 only dilutes ^13^C accumulation in citrate and glutamine from [U-^13^C]βHB metabolism in brain slices. **Table S1.** Intracellular amino acid amounts of incubated cerebral cortical slices. **Figure S3.** Metabolism of βHB is independent of carnitine palmitoyltransferase I (CPT1) in brain slices. **Figure S4.** Inhibition of astrocyte glutamine derived from metabolism of βHB does not affect neuronal GABA synthesis.

## Data Availability

All data of this study is available from the corresponding authors upon request.

## Abbreviations

βHB: β-Hydroxybutyrate
C8: Octanoic acid
C10: Decanoic acid
CPT-1: Carnitine palmitoyltransferase I
GS: Glutamine synthetase
MCFA: Medium-chain fatty acid
MCT: Medium-chain triglyceride
MSO: Methionine sulfoximine
OCR: Oxygen consumption rate
PAG: Phosphate-activated glutaminase
TCA: Tricarboxylic acid
