# Supplementary material for: Astrocyte metabolism of the medium-chain fatty acids octanoic acid and decanoic acid promotes GABA synthesis in neurons via elevated glutamine supply
Source: Mol Brain. 2021 Sep 3;14:132. doi: 10.1186/s13041-021-00842-2 (PMC8414667; doi:10.1186/s13041-021-00842-2)
Supplement: Supplementary file 1 — Additional file 1: Figure S1. Unlabeled 12C-βHB competes poorly with [U-13C]C8 and [U-13C]C10 in brain slices. Figure S2. Unlabeled 12C8 and 12C10 only dilutes 13C accumulation in citrate and glutamine from [U-13C]βHB metabolism in brain slices. Table S1. Intracellular amino acid amounts of incubated cerebral cortical slices. Figure S3. Metabolism of βHB is independent of carnitine palmitoyltransferase I (CPT1) in brain slices. Figure S4. Inhibition of astrocyte glutamine derived from metabolism of βHB does not affect neuronal GABA synthesis. [file 13041_2021_842_MOESM1_ESM.docx]

**
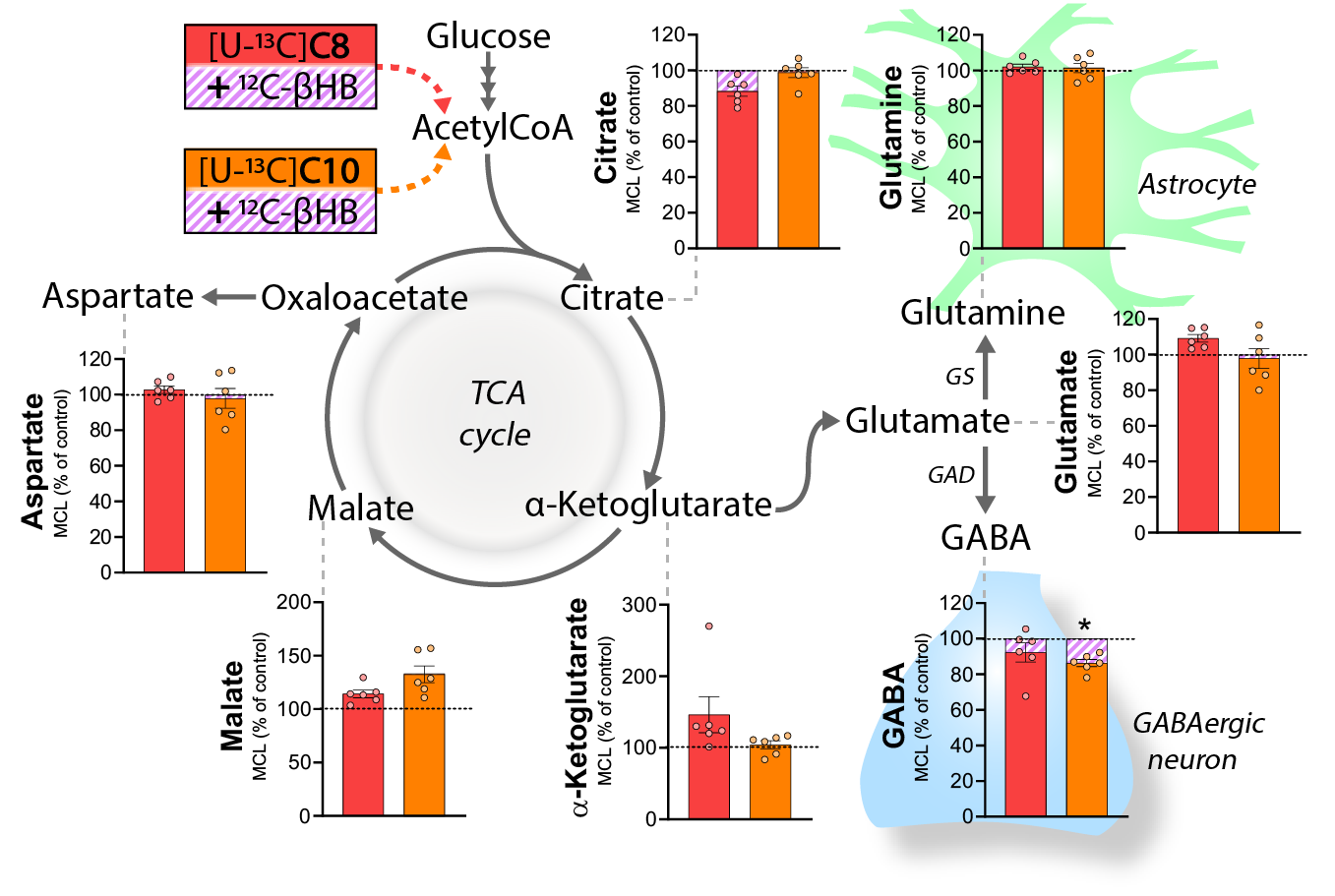
Figure S1: Unlabeled ^12^C-βHB competes poorly with [U-^13^C]C8 and [U-^13^C]C10 in brain slices.**Competition assay between [U-^13^C]C8 + unlabeled ^12^C-βHB and [U-^13^C]C10 + unlabeled ^12^C-βHB in mouse cerebral cortical slices (molecular carbon labeling, MCL, relative to the MCL without ^12^C-βHB (control)). [U-^13^C]C8, [U-^13^C]C10 and ^12^C-βHB were all provided at concentrations of 200 µM in addition to 5 mM D-glucose. Metabolism of an unlabeled substrate (^12^C) will dilute the ^13^C accumulation and hereby lead to a reduction in the MCL. βHB: β-hydroxybutyrate, C8: octanoic acid. C10: decanoic acid, GAD: glutamate decarboxylase, GS: glutamine synthethase. Mean ± SEM, n = 6 from individual animals, repeated measures 1-way ANOVA with Bonferroni post hoc test, *p < 0.05.

***
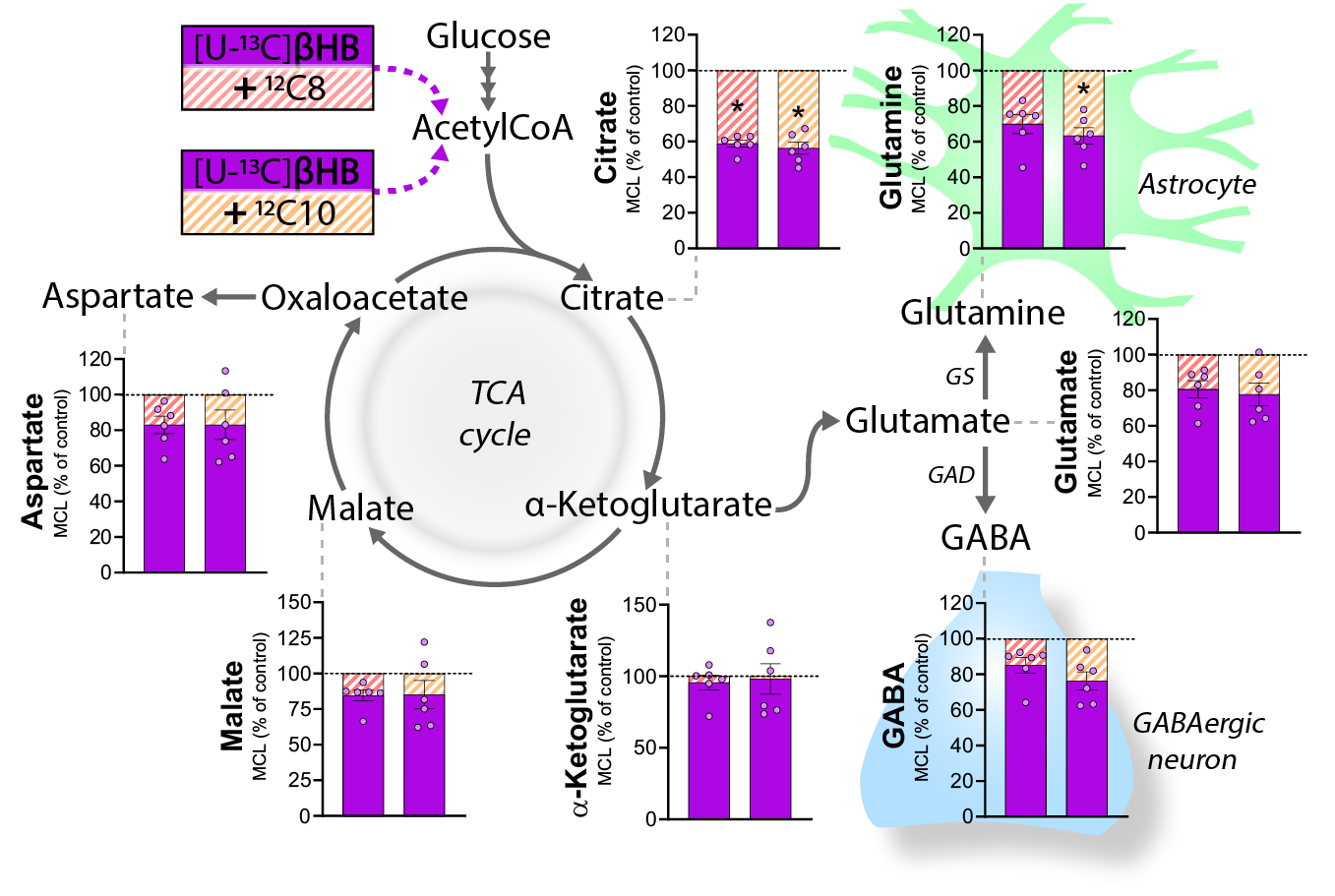
*Figure S2: Unlabeled ^12^C8 and ^12^C10 only dilutes ^13^C accumulation in citrate and glutamine from [U-^13^C]βHB metabolism in brain slices.** Competition assay between [U-^13^C]βHB + unlabeled ^12^C8 and U-^13^C]βHB + unlabeled ^12^C10 in mouse cerebral cortical slices (molecular carbon labeling, MCL, relative to the MCL without competing ^12^C fatty acids (control)). [U-^13^C]βHB, ^12^C8 and ^12^C10 were all provided at concentrations of 200 µM in addition to 5 mM D-glucose. Metabolism of an unlabeled substrate (^12^C) will dilute the ^13^C accumulation and hereby lead to a reduction in the MCL. βHB: β-hydroxybutyrate, C8: octanoic acid. C10: decanoic acid, GAD: glutamate decarboxylase, GS: glutamine synthethase. Mean ± SEM, n = 6 from individual animals, repeated measures 1-way ANOVA with Bonferroni post hoc test, *p < 0.05.

| Amino acid amounts (nmol/mg) | **C8** | **C10** | **βHB** | **C8+C10** | **C8+βHB** | **C10+βHB** | ANOVA  p-value | Multiple  comparisons |
| --- | --- | --- | --- | --- | --- | --- | --- | --- |
| **Aspartate** | 59.2±7.5 | 36.5±3.9 | 36.4±8.9 | 49.1±9.0 | 47.2±7.5 | 34.0±2.8 | 0.2020 | *n/a* |
| **Glutamate** | 178.3±21.1 | 119.8±9.8 | 100.2±24.7 | 183.8±19.0 | 231.5±46.0 | 153.9±9.0 | 0.0503 | *n/a* |
| **Glutamine** | 16.8±2.2 | 10.7±1.1 | 11.1±2.7 | 20.7±2.5 | 20.6±4.4 | 14.5±1.4 | 0.0841 | *n/a* |
| **Taurine** | 61.8±7.6 | 42.1±3.7 | 44.1±10.9 | 62.1±7.5 | 65.5±8.4 | 55.9±2.2 | 0.2096 | *n/a* |
| **GABA** | 20.1±2.5 | 13.4±0.9 | 11.6±3.2 | 25.5±3.1 | 23.1±3.7 | 17.5±1.0 | **0.0432*** | *n.s.* |

**Supplementary table 1: Intracellular amino acid amounts of incubated cerebral cortical slices.**βHB: β-hydroxybutyrate, C8: octanoic acid. C10: decanoic acid, n/a: not applicable, n.s.: not significant. Mean ± SEM, n = 6 from individual animals, repeated measures 1-way ANOVA with Bonferroni post hoc test, *p < 0.05.

**
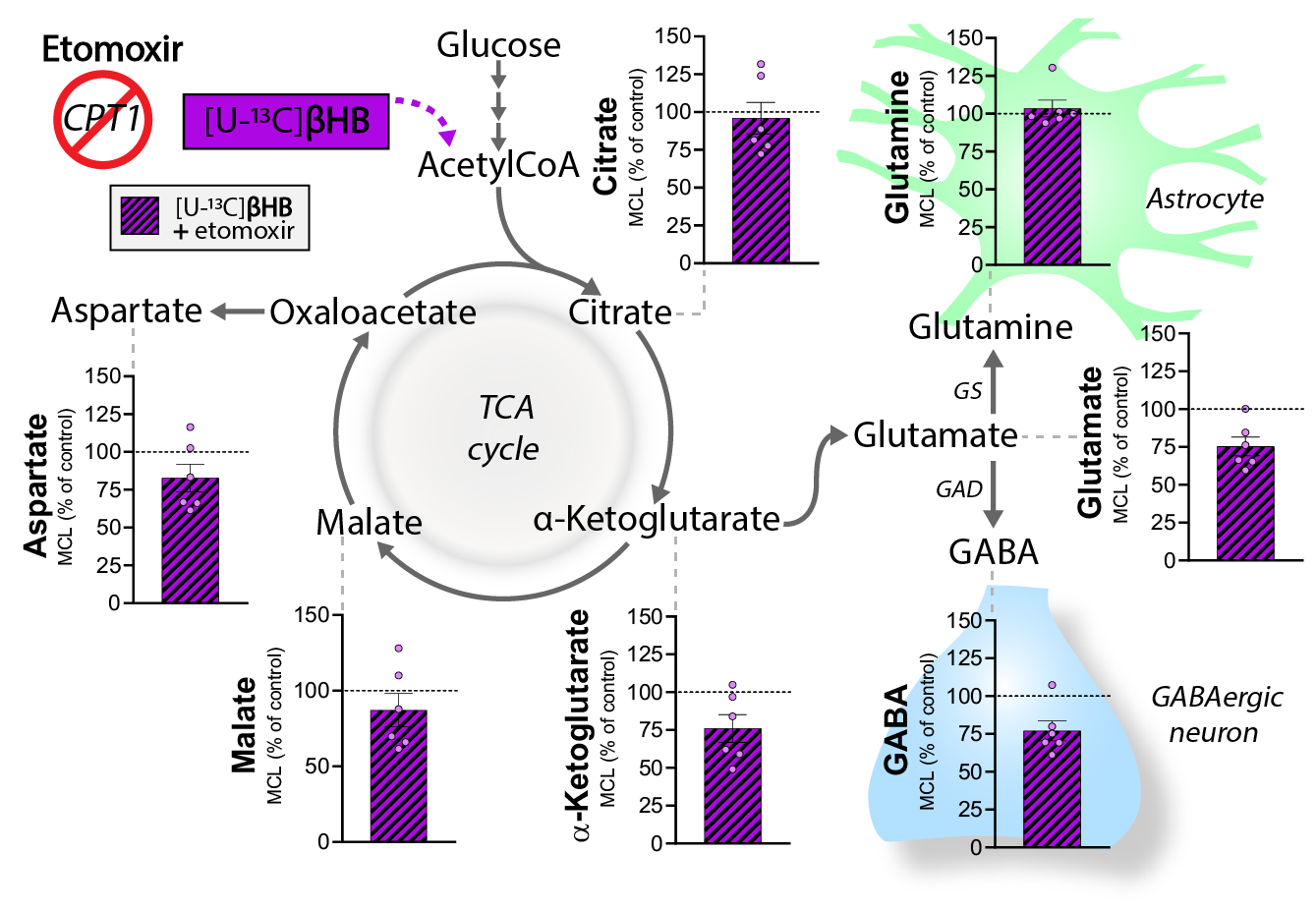
Figure S3: Metabolism of βHB is independent of carnitine palmitoyltransferase I (CPT1) in brain slices** Metabolism of [U-^13^C]βHB (purple/black striped bars) in the presence of the CPT1 inhibitor etomoxir in mouse cerebral cortical slices (molecular carbon labeling, MCL, relative to the MCL without etomoxir (control)). [U-^13^C]βHB was provided at a concentration of 200 µM in addition to 5 mM D-glucose. Etomoxir was applied at 100 µM. βHB: β-hydroxybutyrate, GAD: glutamate decarboxylase, GS: glutamine synthethase. Mean ± SEM, n = 6 from individual animals, repeated measures 1-way ANOVA with Bonferroni post hoc test, *p < 0.05.


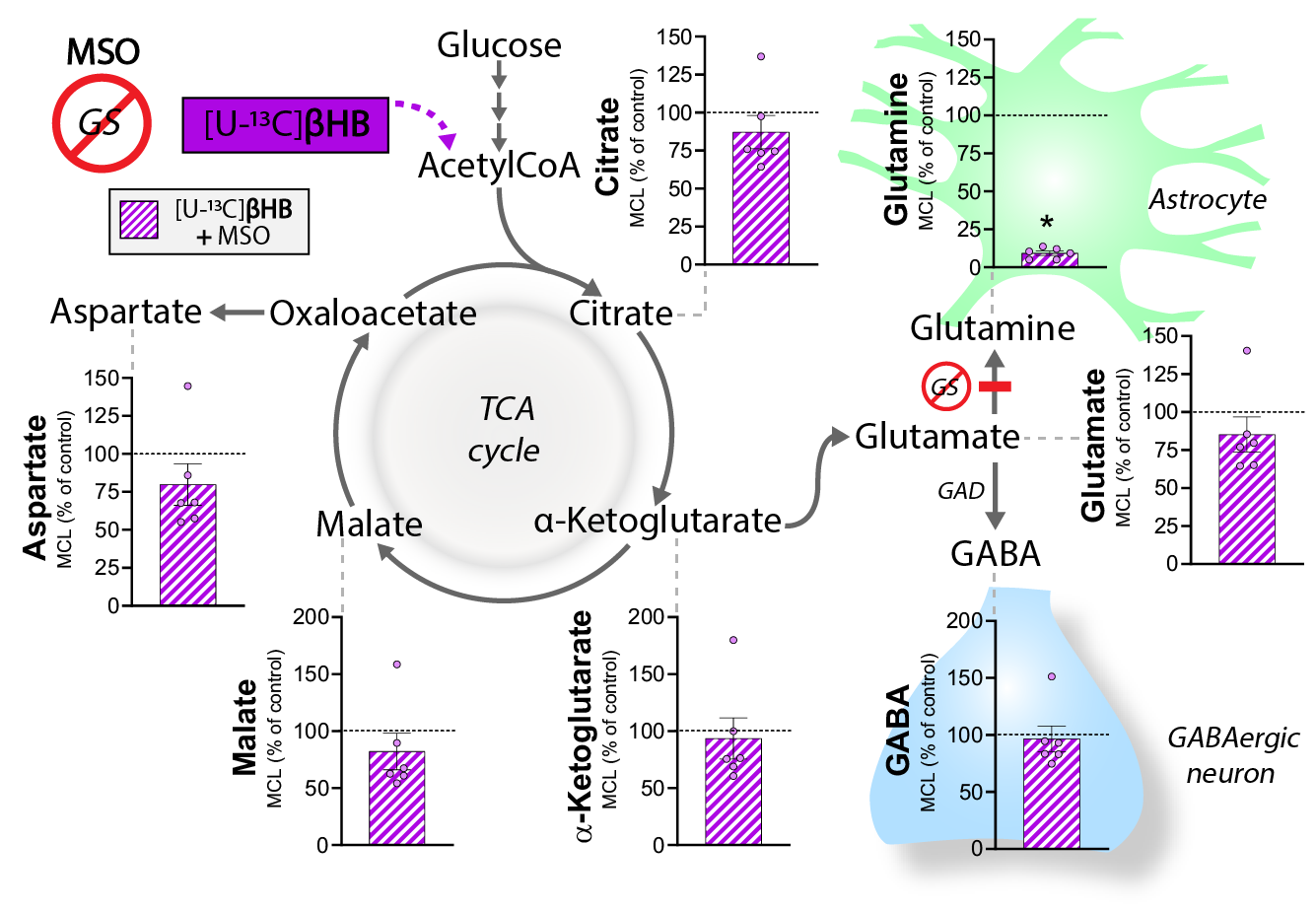
**Figure S4: Inhibition of astrocyte glutamine derived from metabolism of βHB does not** **affect neuronal GABA synthesis.** Metabolism of [U-^13^C]βHB (purple/white striped bars) in the presence of the glutamine synthetase (GS) inhibitor MSO in mouse cerebral cortical slices (molecular carbon labeling, MCL, relative to the MCL without MSO (control)). [U-^13^C]βHB was provided at a concentration of 200 µM in addition to 5 mM D-glucose. MSO was applied at 5 mM. βHB: β-hydroxybutyrate, GAD: glutamate decarboxylase, GS: glutamine synthethase. Mean ± SEM, n = 6 from individual animals, repeated measures 1-way ANOVA with Bonferroni post hoc test, *p < 0.05.
